# Universality and Normativity of the Attachment Theory in Non-Western Psychiatric and Non-Psychiatric Samples: Multiple Group Confirmatory Factor Analysis (CFA)

**DOI:** 10.3390/ijerph18115770

**Published:** 2021-05-27

**Authors:** Naser Abdulhafeeth Alareqe, Samsilah Roslan, Sahar Mohammed Taresh, Mohamad Sahari Nordin

**Affiliations:** 1 Department of Psychology and Counseling, Taiz University (TU), Taiz 6803, Yemen; naser.abdulhafeeth@lms.mediu.edu.my; 2 Department of Foundations of Education, Faculty of Educational Studies, Universiti Putra Malaysia (UPM), Selangor 43400, Malaysia; sahartaresh@yahoo.com; 3 Department of Educational Psychology and Counseling, International Islamic University Malaysia (IIUM), Selangor 53100, Malaysia; msahari@iium.edu.my

**Keywords:** attachment orientations, attachment psychosis, multiple group CFA

## Abstract

This study tests for the first time the validity of universality and normativity assumptions related to the attachment theory in a non-Western culture, using a novel design including psychiatric and non-psychiatric samples as part of a comprehensive exploratory and advanced confirmatory framework. Three attachment assessments were distributed to 212 psychiatric outpatients and 300 non-psychiatric samples in Yemen. The results of the fourteen approaches of exploratory factor analysis (EFA) produce a similar result and assertion that the psychiatric outpatients tend to explore attachment outcomes based on multi-methods, while the non-psychiatric samples suggest an attachment orientation based on multi-traits (self–other). The multiple group-confirmatory factor analysis (MG-CFA) demonstrates that the multi-method model fits the psychiatric samples better than the non-psychiatric samples. Equally, the MG-CFA suggests that the multi-traits model also fits the psychiatric samples better than the non-psychiatric samples. Implications of the results are discussed.

## 1. Introduction

The attachment theory originated with John Bowlby [1,2,3]. He practiced psychoanalysis with 44 juvenile thieves who exhibited psychopathic disorder symptoms [1,4]. Through his observations of these patients, Bowlby discovered that there were some fundamental problems in the ways the clients perceived and behaved in relationships. He theorized that the development of these problems had to have occurred early in their childhood. Bowlby [3] maintained that in organizing individual and subjective experiences, they had constructed internal working models, such as mental representations of the self and others.

### 1.1. Child–Mother Relationship and Internal Working Models of Self (Anxiety) and Other (Avoidance)

According to the attachment theory, during infancy a bond is formed between the infant and mother. If the child receives sensitive and supportive care in these early years, with the parents emotionally and physically available, the child will develop a sense of security. Others in the environment can also provide the necessary support and caregiving if the attachment figure is unavailable. If the child does not receive sensitive and supportive caregiving, then the child will develop deficiencies in feelings about the self and others, as well as emotional reactions, such as depression, anxiety, and fear. This early attachment lays the foundation for future relationships. It is the individual’s subjective experience of others in relationships which is of crucial importance [3,4]. Bowlby’s work emphasizing the quality of early adaptation and continuity in experience provides a framework for conceptualizing early relationship disturbances and their links to psychopathology [5].

Bowlby [3] maintained that in organizing the individual’s subjective experiences, she or he constructs internal working models, namely, mental representations of the self and others. According to Bowlby [3]: “Every situation we meet within life is construed in terms of the representational models we have of the world about us and of ourselves. Information reaching us through our sense organs is selected and interpreted in terms of those models, its significance for us and for those we care for is evaluated in terms of them and plans of action conceived and executed with those models in mind. On how we interpret and evaluate each situation, moreover turns also about how we feel” (p. 229).

Additionally, Bowlby suggested that infants begin to develop internal working models by the second half of the first year. Children who have received sensitive and supportive caregiving by caregivers construct secure models of relationships as gratifying and dependable, as well as secure models of themselves as worthy of affection and attention. Children who have experienced insensitive and maternal deprivation develop insecure internal working models of others as uncaring and non-dependable, and of self as undeserving.

While Bowlby was the primary author of attachment theory, others have made important contributions to the field of developmental psychopathology based on his concepts. Mary Ainsworth was one of Bowlby’s students and colleagues and eventually became known as the methodologist behind attachment theory research. She studied with Bowlby for a time and later went on to conduct research that supported and refined attachment theory [5].

### 1.2. Child Attachment in Non-Western Culture

Bowlby (1969/1982) asserted that attachment is indeed a universal phenomenon. Thus, cross-cultural studies on child attachment have burgeoned [1,2]. The scope of research on childhood attachment is to explore the four assumptions of attachment theory: universality, normativity, sensitivity, and competence.

The cross-cultural studies on childhood attachment supported Bowlby’s (1969/1982) idea of “attachment universal phenomenon”. For example, van IJzendoorn and Sagi [2] conducted a meta-analysis drawn from several different studies among non-Western cultures (Africa, China, Israel, Japan, and Indonesia) compared with Western Europe and the United States. The results demonstrated that the four core assumptions of attachment theory on childhood studies confirmed the universality of attachment theory. The universality assumption appeared to be strongly supported in that similar patterns of attachment behavior have been observed in every cross-cultural study. The cross-cultural evidence for the normativity assumption is rather strong as well in that the majority of infants were classified as securely attached in all cross-cultural studies. The sensitivity and competence assumptions received less support due to the lack of statistical power analysis, effect size, and small sample size. A recent meta-analysis of more than 65 studies showed that the association between attachment and sensitivity is important but modest.

### 1.3. Adult Attachment Models

#### 1.3.1. Hazan and Shaver’s Categorical Measure of Attachment

The first model of adult attachment, which has generated interesting research, was developed by Hazan and Shaver [6]. Their model is a translation of the Ainsworth et al. classification into categories appropriate for adults. This model conceptualizes adult close relationships within the framework of attachment theory in terms of the internal working model (IWM) generated from child–parent interaction in childhood.

Hazan and Shaver [6] suggested that a close relationship can be conceptualized as an attachment process. Emotional bonds in adulthood are formed between two adults (adult–adult relationships), whereas earlier in life they are formed between infants and primary caretakers. The relation between infant and adult attachment processes can be explained in terms of the continuity of attachment styles due to “inner working models” of the self and significant others [6,7]. These working models guiding expectations and behaviors are significant in the maintenance and continuity of attachment styles.

Hazan and Shaver [6] examined how well Bowlby and Ainsworth’s conceptual models of attachment fit with the concepts of adult romantic love, loneliness, depression, and grief in a sample of participants (age = 36) who responded to a newspaper article on love styles and who completed an accompanying questionnaire. One hypothesis was that adults would fall into categories of attachment similar to those developed by Ainsworth and colleagues. They developed a categorical measure that asked individuals to place themselves in one of three descriptive categories (secure, avoidant, or anxious/ambivalent) after reading three brief styles (see Table 1).

Hazan and Shaver [3] found that the percentages of adults who classified themselves as secure (56%), avoidant (25%), and anxious/ambivalent (19%) were similar to those reported by Ainsworth et al. [4] in a sample of infants as 62%, 23%, and 15%, respectively. Hazan and Shaver [3] conducted another survey of their study with a college sample because they suspected limitations in the earlier study, such as the possibility of biased results since the data consisted of subjects who volunteered to participate in the study, as well as the neglect of mental models of the self due to space limitations in the newspaper.

Similar proportions were obtained among the three attachment styles (56% secure, 23% avoidant, and 20% anxious/ambivalent). The similar proportions of attachment styles found in both of these studies when compared to Ainsworth et al. may indicate both the reliability and continuity of these attachment styles from childhood to adulthood. Since Hazan and Shaver’s [3] seminal work, adult attachment theory has been used in the evaluation of several phenomena including work, marriage, leadership, and adult psychopathology [5].

#### 1.3.2. Four-Factor Model of Adult Attachment (Bartholomew, 1990)

The third model of adult attachment, proposed by Bartholomew [8], is an extension of Bowlby’s notion that there exist mental models of the self and others that guide expectations and behavior [3].

##### Conceptualization

Bowlby’s concept of internal working models provided ground for the growing interest in the continuity of attachment patterns from infancy into childhood and adulthood [6,9].

Bartholomew [8] agreed with Bowlby that internal working models operate during childhood to develop attachment patterns, and throughout adolescence and adulthood to maintain them. She also agreed with adult attachment researchers [7,10] that adult attachment is a significant area to study and learn more about a variety of human behaviors. Bartholomew’s theoretical and empirical work focused not on infants or children but attachment in adults. When a particular relationship is specified, it is with a friend or romantic partner rather than with a parent. Since these relationships are believed to function in such a way that each partner may serve as an attachment figure to the other, they are frequently referred to as indicating “reciprocal” attachment [11,12].

Based on Bowlby’s theoretical assumption of internal models of the self and others, Bartholomew and Horowitz [13] suggested that dichotomizing each model of the self and others into positive or negative yields four different attachment styles. This four-factor model was the first study to examine the four styles derived from a combination of two dimensions: two levels of self-image (positive or negative), and two levels of the image of others (positive or negative).

##### Four-Factor Model

**Figure 1:**
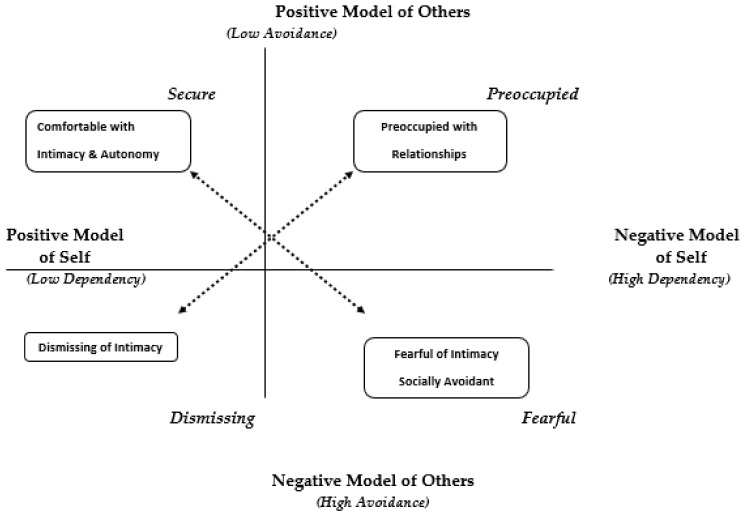
Bartholomew’s (1990) Four-Factor Model of Adult Attachment.

Again, Bartholomew [14] assumed that adult attachment might better be conceptualized as a four-group classification (Figure 1), with two underlying axes as described here.

**Secure Style:** According to Bartholomew and Horowitz [15], a positive mental model of the self and others yields a secure attachment style, namely, a sense of worthiness and an expectation that people are generally accepting and responsive. Securely attached people have generally high self-esteem and an absence of serious interpersonal problems. Secure individuals have an internalized sense of self-worth [6], sometimes called “lovability”, and are comfortable with intimacy in close relationships. They expect others in relationships to be generally accepting and responsive [7,14,15].

**Preoccupied Style:** Bartholomew and Horowitz [13] indicated that a negative mental model of the self and a positive mental model of others yields a preoccupied attachment style: a sense of self-unworthiness, but a positive evaluation of others. Preoccupied individuals tend to feel unloved and unlovable, and at the same time hold a generally positive view of others. This combination creates an intense desire to gain approval as if to validate some modicum of self-worth by gaining the acceptance of others who are valued.

In this style, an individual acquires a sense of self-acceptance as a direct result of being accepted by others who are significant to that individual. Bartholomew and Horowitz also stated that this style corresponds to Hazan and Shaver’s anxious-ambivalent type. Their deep-seated sense of unworthiness motivates them to strive for excessive closeness in personal relationships and to interact in a rather dependent fashion [8,13].

**Fearful Style:** A negative mental model of the self and others yields a fearful-avoidant attachment style—a sense of unworthiness and an expectation that people are generally untrustworthy and rejecting. Fearfully attached individuals have a negative self-image. Like the preoccupied group, they have an intense sense of unworthiness (or “unlovability”) that makes them highly dependent on others for their self-worth. However, due to their generally negative view of others, they additionally experience chronic distrust and fear of rejection.

Since they expect others to be untrustworthy and rejecting, they avoid close involvement with others. Although they desire contact and intimacy, they nonetheless shun it, to preclude the pain of (anticipated) rejection and loss [7,14,15,16]. Bartholomew and Horowitz [15] suggested that this category may be the avoidant style as identified by Hazan and Shaver [3].

**Dismissing Style:** Finally, a positive mental model of the self and a negative mental model of others yield a dismissive attachment style: a sense of worthiness and an expectation that people are generally untrustworthy and rejecting. Dismissive individuals, like fearful ones, avoid closeness in relationships because of their negative expectations of others. However, they generally have a positive view of themselves as being worthy and lovable. They avoid intimacy with others to protect their self-image.

This avoidance prevents the experience of negative affect and the arousal of attachment behaviors. Even when overtly rejected by an attachment figure, this denial defensively allows them to maintain a positive sense of self. They tend to view relationships as unimportant and prize their independence from others. They create a sense of invulnerability by rejecting others before they can be rejected [7,14,15,16].

#### 1.3.3. Two Dimensions of Dependency and Avoidance

Bartholomew [14] also conceptualized the four styles of attachment in terms of dependency on others for positive self-regard (low to high) and avoidance of intimacy (low–high). The secure style is characterized by low dependency on others for high self-esteem and positive self-regard and by avoidance of intimacy.

The dismissing style is also characterized by low dependency on others for positive self-regard, but with high avoidance of intimacy. That is, they are independent and autonomous but lack intimate relationships. The preoccupied style is characterized by high dependency on others for positive self-regard and self-approval combined with low avoidance of intimacy. The fearful style is characterized by high dependency on others for positive self-regard as well as high avoidance of intimacy; they have neither autonomy nor intimacy in interpersonal relationships.

#### 1.3.4. Two-Factor Model: Anxiety and Avoidance (Brennan et al., 1998)

**Figure 2:**
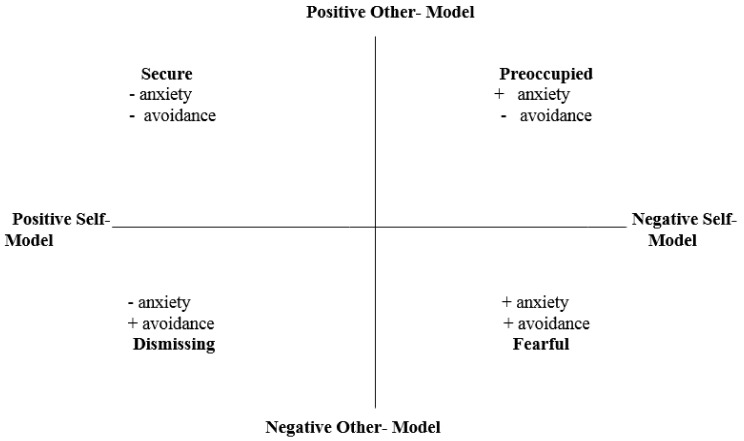
Four-Factor Model of Adult Attachment (Bartholomew & Griffin, 1994) with Two-Dimensional Model of Attachment (Brennan, Clark, & Shaver, 1998) Overlaid.

In response to the creation of adult attachment instruments, Brennan, Clark, and Shaver [17] constructed an integrated measure by using all of the non-redundant items from all published (and even some unpublished) adult attachment instruments. This resulted in a 323-item instrument which was administered to 1086 college students. A factor analysis revealed two primary factors, similar to Bartholomew’s two primary dimensions, which accounted for 62.8% of the total variance. Brennan and her colleagues labeled these factors as anxiety and avoidance (Figure 2). A hierarchical cluster analysis also revealed four categories which are parallel with Bartholomew’s four categories of secure, preoccupied, dismissing, and fearful. Individuals with low anxiety and low avoidance were classified as “secure”. Those who were low anxiety and high on avoidance were classified as “dismissing”. Subjects who were high on anxiety and low on avoidance were placed in the “preoccupied” category and those who were high on both anxiety and avoidance were classified as “fearful” [17].

Briefly, two dimensions have been found to underlie self-report measures (Brennan et al., 1998), which can be conceptualized in cognitive terms as ‘model of self’ and ‘model of other’; or in affective and behavioral terms as ‘anxiety’ and ‘avoidance’ [18]. Attachment anxiety (model of self) is associated with a negative self-image and an excessive need for approval from others, coupled with a fear of rejection and abandonment. Attachment avoidance (model of other) is associated with a negative image of others and is defined in terms of either an excessive need for self-reliance or fear of depending on others.

#### 1.3.5. Two-Factor Model: Anxiety and Avoidance (Berry et al., 2006)

Brennan, Clark, and Shaver [17] developed a two-factor model (anxiety and avoidance) based on Bartholomew’s four-factor model. The two-factor model focused on close and general relationships (e.g., close friends and family members). More recently, Berry and her colleagues [19] claimed that attachment theory had limitations in assessing attachment styles in psychosis.

Subsequently, they developed a new measurement model to assess adult attachment styles based on Bartholomew’s model [20] and adapted it for use with individuals with psychosis. Psychosis in Berry’s model is associated with interpersonal difficulties and low self-esteem [21]. Interpersonal difficulties include aggression, poor sociability, and excessive dependence on others. This model contains two factors, psychosis anxiety and psychosis avoidance [22], assessed by the Psychosis Attachment Measure (PAM) [19] and reflecting psychotic phenomena in its items. Its content refers to thoughts, feelings, and behaviors in interpersonal difficulties without referring to specific relationships [23].

#### 1.3.6. Conclusion

Through his experiences with juvenile thieves whom he categorized as “antisocial clients”, Bowlby formulated his tenets about personality (child–mother relationship and internal work model). Based on Bowlby’s concepts (secure versus insecure), Ainsworth designated strange situation assessment, creating three different patterns of childhood attachment [secure, avoidant, and anxious/ambivalent]. With her contribution, attachment theory has become an empirical conceptualization. Hazan and Shaver [6] applied Ainsworth’s attachment classifications in adulthood, yielding similar results.

Bartholomew [20] yielded that the four attachment styles in adults can be identified as ‘secure’ (analogous to secure attachment styles of children), ‘dismissing’ (avoidant in children), ‘preoccupied’ (resistant, anxious, ambivalent, or anxious/ambivalent in children), and ‘fearful’ (disorganized in children). Brennan, Clark, and Shaver [17] found two orthogonal dimensions (avoidance and anxiety) under Bartholomew’s four categories of adult attachment. More recently, Berry et al. [19] developed a new measurement model (anxiety and avoidance) based on Bartholomew’s four-factor model [20]. The merit of this model is related to psychotic phenomena in clinical and non-clinical groups.

### 1.4. Attachment Theory and Therapy

In terms of therapy, the attachment theory has helped create important interventions for psychiatric disorders. However, therapeutic programs cannot be limited to those relevant to the attachment theory in clinical and non-clinical settings and have to include other therapeutic programs relevant to the other schools of psychology. In this respect, Obegi and Berant [24] discussed the four programs based on the attachment theory outlined by European clinicians which are attachment-based therapy [8], attachment-based psychoanalytic psychotherapy [9], attachment narrative therapy [10], and the attachment guided approach established by Brisch [11].

Additionally, self-reports on attachment can be used to measure attachment orientations early in the treatment and to track changes over time in psychiatric patients for whom insecure attachment orientations are clinically relevant [12,13,20]. In addition, projective tests are deemed highly useful in exploring the unconscious correlates of self-reports of attachment orientations [25].

### 1.5. Assumptions of Attachment Theory

Theoretically, the attachment theory assumes universality, which means that the attachment models are prevalent in every culture and fit all abnormal (e.g., psychiatric patients, psychiatric outpatients, and forensic population) and normal (e.g., students and community) settings. Consistent with the normativity assumption of the attachment theory, the insecure attachment models are more prevalent and fit in abnormal rather than normal settings [14]. Accordingly, the model attachments (e.g., psychosis attachment and attachment dimensions) and self–other traits fit both psychiatric and non-psychiatric groups; however, the psychiatric group scores higher than the non-psychiatric group.

At the onset, Bowlby [26] and others [16] assumed that the attachment theory was universal and possessed culturally invariant applicability. This meant that the assumptions of the attachment theory, particularly universality and normativity, were expected to be equivalently prevalent in all cultures. Van Ijzendoorn and Sagi [15] conducted a meta-analysis drawn from several different studies conducted in the context of non-Western cultures (Africa, China, Israel, Japan, and Indonesia) compared with those conducted in Western Europe and the United States. The results demonstrated that the assumptions of the attachment theory studies confirmed the universality of the attachment theory. The universality assumption appears to be supported strongly in that similar patterns of attachment behavior have been observed in every cross-cultural study. The cross-cultural evidence for the normativity assumption is rather strong, with the majority of infants being classified as securely attached in all cross-cultural studies.

However, the critics of the theory [27,28] argue that the attachment theory is not applicable to other cultures since it is deeply rooted in Western cultural values, especially since research supporting the attachment theory is based primarily on Western cultures. The limited studies conducted in non-Western cultures either supported, rejected, or found these assumptions unexamined [28,29]. Some studies [9,30,31] supported the universality and normativity assumptions while other studies [29,32,33,34,35] rejected these assumptions. Given this conflicting body of evidence, Cassidy and Shaver [36] noted that the data on attachment in the Muslim cultural context are still lacking.

### 1.6. Aims of the Study

The general purpose of the study is to validate the universality and normativity assumptions of the attachment theory in both psychiatric and non-psychiatric groups from a non-Western context. Specifically, the aims of the study are to test the normal distribution of the eight attachment outcomes in psychiatric and non-psychiatric groups, to explore the attachment outcomes, and to examine multi-methods of three attachment scales as well as multi-traits of the self–other in the attachment theory in both psychiatric and non-psychiatric groups.

## 2. Methods

### 2.1. Participants

#### 2.1.1. Psychiatric Sample

A questionnaire packet was administered by the researcher himself through a semi-structured interview with each patient in the Psychiatric Hospital of Taiz City and two private psychiatry clinics in Yemen after obtaining the consent of the participants. Four months were spent to meet the optimum sample size (212 psychiatric outpatients). This sample size is a sufficient number for comparing the multiple groups of CFA (Kline, 2016). The average age of the psychiatric group was 30.81 and the standard deviation was 7.32. Data show that 56.63% of participants were married, 34% were single, and 9.4% were divorced. Approximately 86.3% of the sample were male. About 28.3% of the sample had basic education (writing and reading skills), 23.1% had primary education, 35.4% held secondary school certificates, and 13.2% were degree holders. Using a non-probabilistic sampling method, the purposive sampling was used to reach 212 outpatients. The inclusion criteria for participants were: (a) Yemeni nationality, (b) being more than 18 years old and above, and (c) getting medical treatment in a psychiatric clinic for more than one year.

#### 2.1.2. Non-Psychiatric Sample

The questionnaire packet for undergraduate students from Taiz University in Yemen was administered by a professional team (the researcher himself, a professor of counseling and guidance, a lecturer of psychology, and a PhD candidate in psychology) under actual classroom conditions. The team gave extra credit to the students who were taking introductory psychology courses, such as theories of personality, theories of learning, and introduction to psychology. These courses were offered to all students of the Faculty of Education. Of the 439 valid cases, 300 cases were randomly chosen to equate the sample size of the clinical sample. The mean age of the non-psychiatric group was 22.76 with a standard deviation of 1.488.

### 2.2. Study Design

This study was classified as correlational design using the analysis of correlation and covariance matrix as the main analyses. Based on this design and main analysis, the exploratory and confirmatory factor analyses with multiple groups were used [37].

### 2.3. Measurements

#### 2.3.1. Relationship Scales Questionnaire (RSQ)

The Relationship Scales Questionnaire (RSQ) consists of a 30-item questionnaire based on the four-category model of adult attachment [38]. Participants rate each item on a 5-point Likert scale reflecting the degree to which each item describes their attachment categories, which include secure, fearful, preoccupied, and dismissing attachment styles.

Schafer and Bartholomew [38] provided the RSQ psychometric properties of the two-dimension questionnaire, the view of self and the view of others, that underlie these four attachment styles. The convergent and discriminant validity of these two dimensions is demonstrated using multi-trait and multi-method matrices and confirmatory factor analyses.

#### 2.3.2. Experiences in Close Relationship Scale (ECRS)

The Experiences in Close Relationship Scale (ECRS) constitutes a 36-item self-report measure of adult attachment [17]. It was developed from over 1000 undergraduate student responses to 323 items representing more than 60 adult attachment subscales. The ECRS was used to assess scores on two relatively orthogonal dimensions underlying anxiety attachment (self-model) and avoidance attachment (other-model).

Using the psychiatric sample, the internal consistency coefficient for each dimension was calculated resulting in the value of alpha of 0.93 for avoidance attachment and 0.89 for anxiety attachment [39]. Furthermore, Brennan, Clark, and Shaver [17] reported coefficient alphas of 0.91 and 0.94, respectively for the anxiety and avoidance subscales. The authors reported a test–retest reliability (3-week period) of 0.70 for two subscales.

Brennan and Shaver [40] reported the preliminary evidence supporting the validity of the ECR, which included the concurrent validity as characterized by strong correlations with self-report attachment measures.

#### 2.3.3. Psychosis Attachment Measure (PAM)

The Psychosis Attachment Measure (PAM) [38] consists of a more recent measurement model of the attachment theory. The items were derived from existing self-report attachment measures [17] yet specific items were referring to psychosis attachment (e.g., “I try to cope with stressful situations on my own”).

The PAM has 16 items of which eight items assess the construct of avoidance and eight items assess the construct of anxiety. The total scores were calculated for each dimension by averaging individual item scores, with higher scores reflecting higher levels of anxiety and avoidance. Berry suggested good reliability for the PAM, with alphas of 0.96 for the anxiety subscale and 0.85 for the avoidance. Moreover, the PAM has been shown to have good psychometric properties in two independent non-clinical samples [19,41,42,43,44]. The study was ethically approved by the Committee of Department of Educational Psychology and Counseling in International Islamic University Malaysia (IIUM), Selangor, Malaysia.

### 2.4. Statistical Analysis

#### 2.4.1. Normal Distributions Techniques

Skewness <1 and kurtosis <1 index in SPSS Statistics 22 are used to test the normal distribution of attachment outcomes. Skewness <1 and kurtosis <1 indices are good techniques for normal distribution [18]. Furthermore, mean and median are employed to the normal distribution of attachment outcomes. Identical values for mean and median are an indicator for normal distribution, while values of the mode are not highly different with mean and median [19].

#### 2.4.2. Exploratory Factor Analysis (EFA)

Seven extractions of the exploratory factor analysis (EFA) were adjusted by orthogonal and oblique rotations creating the fourteen results for attachment outcomes. Using several methods of the exploratory factor analysis can give more confidence and ensure the high validity for data while obtaining similar results [18].

#### 2.4.3. Confirmatory Factor Analysis (CFA)

The *p*-value in the Chi-square (χ^2^) model tends to be statistically insignificant. However, it can be significant using a large sample size, a small number of model variables, and big coefficients of correlation [45,46]. Normed Chi-square (χ^2^) must be <3. Although the comparative fit index (CFI) and incremental fit index (IFI) ≥90 are acceptable [45,47], Hu and Bentler [48] suggested that CFI and IFI ≥95 create an excellent fit for the model’s data. Although both squared root mean residual (SRMR) and root mean square error approximation (RMSEA0 ≤0.08) are recommended [45,46,47], values of SRMR and RMSEA ≤0.05 demonstrate an excellent fit for the model’s data [48].

#### 2.4.4. Multiple Group CFA

To examine the invariant group of CFA in AMOS 22.0.0 (IBM, Armonk, NY, USA), the procedures in this study followed the steps discussed by Byrne [47]. Step 1 consisted of testing the adequacy of the model with pooled data including psychiatric and non-psychiatric samples. Step 2 tested the adequacy of the baseline model for the psychiatric and non-psychiatric samples separately. If the practical difference is in both CFI and IFI ≥0.01, as well as in RMSEA ≥0.015, it can be concluded that there are substantial differences in both groups, which means the hypothesized model provides a better fit in one group. The model with the insignificant *p*-value provided a better fit than its pair with significance [45,47,49].

## 3. Results

### 3.1. Normal Distribution of Attachment Outcomes

#### 3.1.1. Psychiatric Sample

The values of the skewness and kurtosis indices (Table 2) shows that the eight attachment outcomes (secure, dismissing, preoccupied, fearful, anxiety-ECRS, avoidance-ECRS, anxiety-PAM, and avoidance-PAM) in the sample of psychiatric outpatients were less than 1 and close to 0, indicating normal and symmetrical distributions.

Furthermore, the measures of the central tendency were equal, thus indicative of the normal distribution of the data. In addition, Table 2 shows that the mean and median values of the eight attachment outcomes were equal and not substantially different from the mode, confirming the normal distributions of variables from the psychiatric sample. In conclusion, the data of the attachment variables were drawn from a normally distributed population.

#### 3.1.2. Non-Psychiatric Sample

Likewise, the values of the skewness and kurtosis of the eight attachment outcomes in the non-psychiatric sample were less than 1, thus suggesting normal and symmetrical distributions.

Moreover, the measures of the central tendency of the eight attachment indicators were equal, indicating a normal distribution for data from the non-psychiatric sample (Table 2).

In close, the data of the eight attachment variables in the non-psychiatric sample were drawn from a normally distributed population.

### 3.2. Exploratory Factor Analysis

In both psychiatric and non-psychiatric samples, the results of seven extracted methods of the exploratory factor analysis confirmed that the Kaiser-Meyer-Olkin measure of sampling adequacy was 0.733 (>0.60), suggesting that the pattern of correlations was relatively compact, and that factor analysis should produce distinct and reliable factors [50]. Barlett’s test of sphericity reached statistical significance (*p* = 0.000, <0.05), supporting the factorability of the correlation matrix. Anti-image correlation for the attachment outcomes (secure, dismissing, preoccupied, fearful, anxiety-ECRS, avoidance-ECRS, anxiety-PAM, and avoidance-PAM) was >0.60, which is considered an acceptable level. That means that the set of attachment outcomes is suitable for factor analysis.

In the psychiatric sample, using the latent root criterion for retaining factors with eigenvalues greater than 1.0 and the scree plots (figures not shown here), a three-dimension structure was identified with the extracted dimensions explaining 64.402% of the total variance cross seven methods of extraction. The first solution or dimension, adult attachment styles, had an eigenvalue of 2.532 and accounted for 31.651% of the variance. The seven methods of extraction confirmed that this dimension included the four styles of “secure” (loadings mean = 0.468), “dismissing” (loadings mean = 0.703), “preoccupied” (loadings mean = 0.567), and “fearful” (loadings mean = 0.522) (Table 3). The mean of loadings cross fourteen methods of exploratory factor analysis varied from moderate to ideal.

The second solution, attachment dimensions, had an eigenvalue of 1.456 and accounted for 18.205% of the variance. The seven methods of extraction identified that this solution contained the two dimensions of anxiety (loadings mean = 0.895) and avoidance (loadings mean = 0.602). The mean of loadings cross fourteen methods of exploratory factor analysis was an excellent rate.

Finally, as confirmed by the seven methods of extraction, the third solution, attachment psychosis, had an eigenvalue of 1.164 and accounted for 14.546% of the variance. This solution contained the two factors of anxiety (loadings mean = 0.874) and avoidance (loadings mean = 0.624). The mean of loadings cross fourteen methods of exploratory factor analysis was an excellent rate.

In the non-psychiatric sample, using eigenvalues greater than 1.0 and the scree plots (figures not shown here), a two-dimension solution was identified with the extracted dimensions explaining 52.810% of the total variance cross seven methods of extraction. The first solution, self-dimension, had an eigenvalue of 2.532 and accounted for 38.325% of the variance.

The seven methods of extraction confirmed that this solution included three factors: anxiety-ECRS (loadings mean = 0.815), anxiety-PAM (loadings mean = 0.606), and secure (loadings mean = 0.484). The mean of loadings cross fourteen methods of exploratory factor analysis varied from moderate to ideal. The second solution as confirmed by the seven methods of extraction, other-dimension, had an eigenvalue of 1.456 and accounted for 14.485% of the variance. This solution contained four factors: avoidance-ECRS (loadings mean = 0.490), avoidance-PAM (loadings mean = 0.462), dismissing (loadings mean = 0.641), preoccupied (loadings mean = 0.459) and fearful (loadings mean = 0.459) (Table 3). The mean of loadings cross fourteen methods of exploratory factor analysis was rated as adequate.

### 3.3. MG-CFA for Multi-Methods (RSQ, ECRS and PAM)

The result obtained from the psychiatric sample was submitted for the confirmatory factor analysis. Using the pooled sample, the result indicated that the model of multi-methods (RSQ, ECRS, and PAM) needed to improve to reach the adequate goodness indices, which was done between the anxiety of attachment dimension with both anxieties of psychosis attachment and security. The goodness of fit statistics for the baseline model were reasonably excellent (e.g., = 47.328, df = 16, = 2.958, *p* = 0.000, CFI = 0.962, IFI = 0.962, SRMR = 0.0383, and RMSEA = 0.062).

Using separate data, the goodness of fit statistics in the psychiatric group (Figure 1) exhibited excellent fit to the data (e.g., = 24.072, df = 16, = 1.505, *p* = 0.088, CFI = 0.975, IFI = 0.976, SRMR = 0.0409, and RMSEA = 0.049), while the goodness of fit statistics in the non-psychiatric sample (Figure 2) was less than that of the psychiatric group (e.g., = 43.985, df = 16, = 2.749, *p* = 0.000, CFI = 0.948, IFI = 0.949, SRMR = 0.0511, and RMSEA = 0.076).

**Figure 3:**
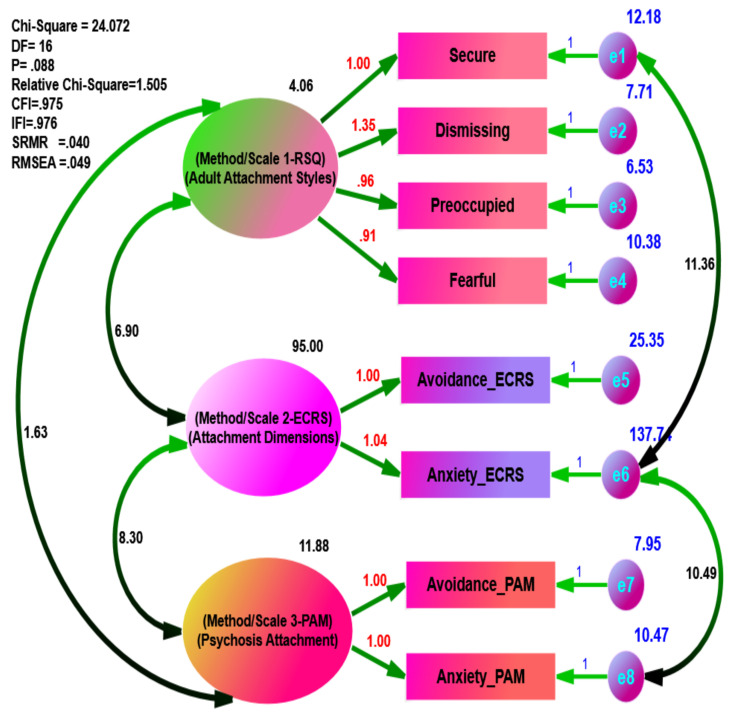
Multi-Methods of Hypothesized Measurement Models of the Attachment Theory in Psychiatric Sample.

**Figure 4:**
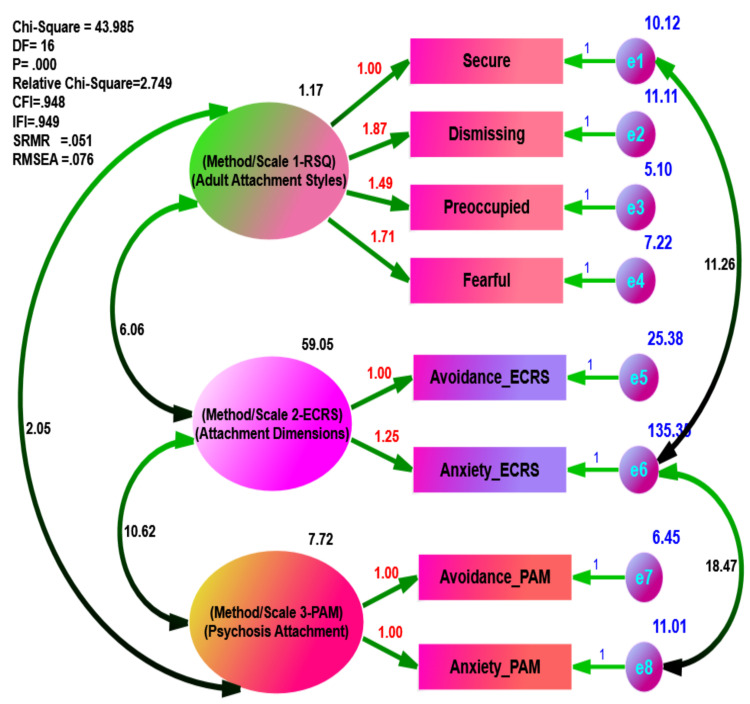
Multi-Methods of Hypothesized Measurement Models of the Attachment Theory in Non-Psychiatric Sample.

Moreover, the *p*-value of the psychiatric group model (*p* = 0.088) was statistically insignificant, thus indicating the hypothesized model of multi-methods (RSQ, ECRS, and PAM) in the attachment theory was a better fit for the psychiatric group than the non-psychiatric group (*p* = 0.000). Additionally, the difference in practical fit (CFI = 0.027 and RMSEA = 0.027) was substantial between the psychiatric and the non-psychiatric group, demonstrating that the hypothesized model of multi-methods in the attachment theory was a better fit for the psychiatric than the non-psychiatric group. For the comparison of the other parameters, the unstandardized estimates were conducted as depicted in Figure 3 and Figure 4.

### 3.4. MG-CFA for Multi-Traits (Self-Other Models)

The result obtained from the non-psychiatric sample was submitted for a confirmatory factor analysis. Using the pooled sample, the result indicated that the model with multi-traits (self–other) needed to be improved to obtain adequate goodness indices, which was done among avoidances and the variables of methods 2 and 3. The goodness of fit statistics for the baseline model were reasonably good and favorable (e.g., χ^2^ = 49.549, df = 16, χ^2^/*df* = 3.097, *p* = 0.000, CFI = 0.959, IFI = 0.959, SRMR = 0.0422, and RMSEA = 0.064).

Using separate data, the goodness of fit statistics in the psychiatric group (Figure 3) exhibited excellent fit to the data (e.g., χ^2^ = 20.191, df = 16, χ^2^/*df* = 1.262, *p* = 0.212, CFI = 0.987, IFI = 0.987, SRMR = 0.043, and RMSEA = 0.035), while the goodness of fit statistics in the non-psychiatric sample (Figure 4) was less than that of the psychiatric group (e.g., χ^2^ = 40.513, df = 16, χ^2^/*df* = 2.532, *p* = 0.001, CFI = 0.955, IFI = 0.956, SRMR = 0.048, and RMSEA = 0.072).

**Figure 5:**
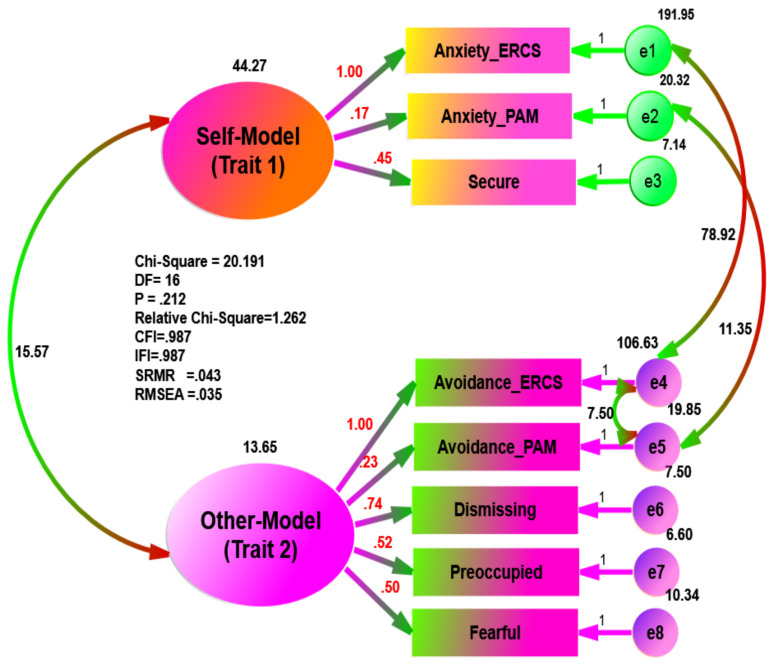
Multi-Traits of Hypothesized Measurement Models of the Attachment Theory in Psychiatric Sample.

**Figure 6:**
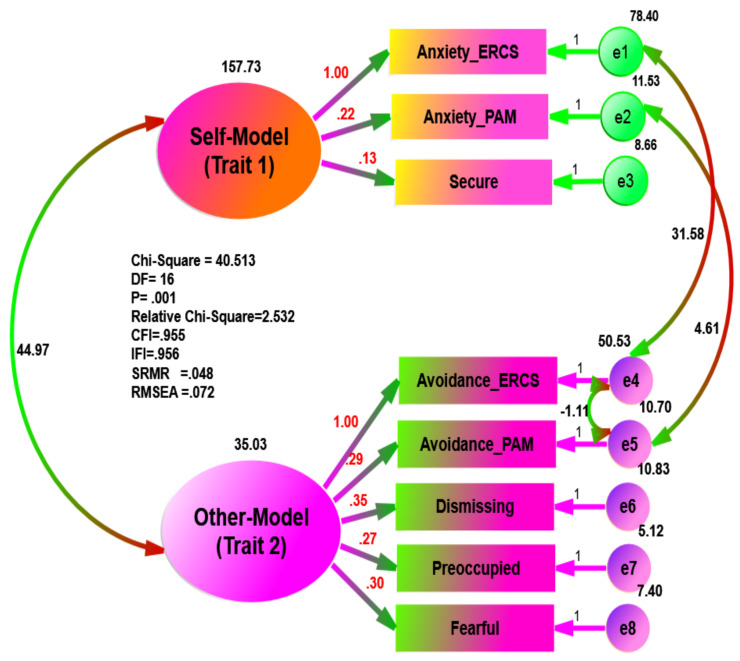
Multi-Traits of Hypothesized Measurement Models of the Attachment Theory in Non-Psychiatric Sample.

Furthermore, the *p*-value of the psychiatric group model (*p* = 0.212) was statistically insignificant, thus indicating the hypothesized model of multi-traits was fitted better for the psychiatric group than the non-psychiatric group (*p* = 0.001). In addition, the difference in practical fit (CFI = 0.032 and RMSEA = 0.037) was substantial between the psychiatric and the non-psychiatric group, thus demonstrating that the hypothesized model of multi-traits in the attachment theory was a better fit for the psychiatric than the non-psychiatric group. For the comparison of the other parameters, the unstandardized estimates were conducted as depicted in Figure 5 and Figure 6. To sum up, this study used three measurements: multi-methods, including different models of attachment, and multi-traits CFA methods which were used based on the self and other.

## 4. Discussion

The general purpose of this study is to evaluate the assumptions of universality and normativity of the attachment theory in cultures different from the original Western culture in whose context it was originally established. Its aims are threefold, and the first is to evaluate the normal distributions of the eight attachment outcomes (secure, dismissing, preoccupied, fearful, anxiety-ECRS, avoidance-ECRS, anxiety-PAM, and avoidance-PAM) in the psychiatric and the non-psychiatric group. The results indicate that the eight attachment outcomes of the attachment theory are normally distributed, confirming the prevalence of attachment orientations in a non-Western, non-psychiatric, and psychiatric sample. Evidently, the obtained results are consistent with the logic of the universality of the original attachment theory and several previous studies. The universality of the attachment theory asserts that all attachment outcomes are prevalent in all cultures and settings [1,2,4,21]. In addition, the previous studies have confirmed that all attachment orientations are available in the cultures and settings in which they are conducted [22,23,51].

The second aim of the study is to explore the eight attachment outcomes in a non-Western, non-psychiatric, and psychiatric sample. The results of the exploratory factor analysis indicate that the psychiatric group resorted to exploring the outcomes of the attachment theory based on multi-methods (RSQ, ERCS, and PAM), while the non-psychiatric sample tended to explain self–other models or anxiety-avoidance as multi-traits of the attachment theory.

The results of both the psychiatric and the non-psychiatric group are in line with the existing studies of the attachment theory [1,2,4,21,22,23,51].

More significantly, the results obtained are identical across the fourteen methods of EFA in both the psychiatric and the non-psychiatric group. Using several statistical methods, the similar results obtained constitute the evidence for the well-structured data, which reflect the validity of the attachment theory in the current study. Developing instruments based on the measurement theory are the strong, and rooted up in reality and literature, results of several extractions of exploratory factory analyses as obtainable, similar, or identical. This statistical evidence is true for different models of attachment theory’s instruments.

The third aim is to examine attachment models based on multi-methods (RSQ, ERCS, and PAM) and multi-traits (self–other) in the psychiatric and non-psychiatric group.

The results of the confirmatory factor analysis indicate that the attachment orientations based on the multi-methods (RSQ, ERCS, and PAM) were reasonably excellent in both sample groups. This is further evidence for the prevalence of the attachment outcomes in both groups.

More significantly, the results of MG-CFA reveal the multi-methods model of the attachment theory is not invariant in both the psychiatric and the non-psychiatric group. Evidently, the results confirm that the multi-methods model fits both sample groups, thus indicating that the model is a better fit for the psychiatric than the non-psychiatric sample group.

The results are in line with the logic of the normativity of the attachment theory, which suggests that the psychiatric group achieves a score higher than that of the non-psychiatric group and that the attachment models are a better fit in the psychiatric than the non-psychiatric group [9,30,31].

Similarly, the results of CFA for the multi-traits (self–other) model show that the attachment outcome was generally favorable in both group samples. This is further evidence for the prevalence of the attachment outcomes (self–other) in the pooled group.

More importantly, the results of MG-CFA indicate that the self–other model of attachment is non-invariant in both the psychiatric and the non-psychiatric group. Prominently, the results prove that the self–other model fits both sample groups, thus demonstrating that the model is a better fit for the psychiatric than the non-psychiatric sample group. The results are in line with the logic of normativity of the attachment theory, which suggests that psychiatric groups score higher than non-psychiatric groups [9,30,31]. That means that the attachment models are a better fit in psychiatric than in non-psychiatric groups.

The consistency between the multi-methods and the multi-traits design is confirmed by the similar results (attachment models in psychiatric groups are a better fit than in non-psychiatric groups), which is the ideal evidence for the validity of normativity of the attachment theory in a non-Western cultural context [9,14,15,16,26,30,31], defeating claims and critics of attachment theory [27,28,29,32,33,34,35].

### 4.1. Implications for Practice

The results confirm that the insecure attachment models fit the psychiatric group sample better than the non-psychiatric group sample. Practically, the psychotherapeutic interventions and therapeutic alliance of the attachment theory are useful for both psychiatric and non-psychiatric groups in non-Western cultural contexts. Moreover, the self-reports of attachment can be used to measure attachment orientations early in treatment and to track changes over time in psychiatric patients for whom insecure attachment orientations are clinically relevant [10].

The therapeutic programs including brief attachment-based therapy [52], attachment-based psychoanalytic psychotherapy [53], attachment narrative therapy [54], and an attachment-guided approach [55] are useful not only in the Western cultural context. The existing literature on attachment orientations suggests that the two projective instruments of the Rorschach and thematic apperception test (TAT) can be used and are useful for exploring the unconscious correlates of self-reports of attachment orientations [51,56,57,58].

Furthermore, therapists and psychotherapists should be more skillful in applying the therapeutic alliance based on the attachment theory in their clinical practice than in their non-clinical practice [51,53,59,60].

### 4.2. Strengths and Limitations

This study used psychiatric and non-psychiatric samples combined with a highly comprehensive analysis to test the assumptions of the attachment theory in the context of a non-Western culture. Sufficiently, the study produced reliable evidence in support of the validity of the universality and normativity assumptions related to the attachment theory in a non-Western culture.

Furthermore, multi-trait multi-methods (MTMM) of CFA constitute an advanced approach for constructing the validity of the theory. Its design requires at least three traits and three methods to obtain reliable results. The separate analysis of the multi-trait multi-methods of CFA is deemed the most appropriate approach in the context of human theories in social psychiatry, public mental health, and psychology that includes two traits such as the self–other model or the internalizing–externalizing model.

On the other hand, the study possessed certain limitations. It focused on confirming the logic of the normativity of the attachment theory and its exact fitness of good indices of analysis, followed by a comparison of each parameter in the psychiatric and non-psychiatric samples. However, Byrne [47] suggested that researchers stop at the stage of the multiple/invariant group of CFA if both groups are not invariant and instead use other techniques such as the multiple indicators multiple causes (MIMIC) modeling.

Next, although the attachment models were validated through psychiatric and non-psychiatric samples, the utilization of therapeutic interventions of the attachment theory still requires further investigations. Moreover, to validate the assumptions of the attachment theory in a non-Western culture, a single study is not sufficient to allow for a generalization of the obtained results that would have to be based on different cultural contexts and settings.

Although this study tested multi-traits and multi-methods of CFA unconnectedly to prove the validity of the attachment theory, future research can use multi-trait multi-methods including self-report attachment measures and adult attachment interviews simultaneously based on optimal sample size (e.g., patients, community, and prisoners). In other words, this study utilized three self-reports of the attachment theory. Another research team on attachment theory has used interviews to collect relevant information such as the adult attachment interview (AAI) [61]. Combining these two approaches into multi-traits and multi-methods can ensure a more sophisticated methodology to test the construct validity for the attachment theory in the non-Western cultural context.

Finally, the attachment theory contains several assumptions such as universality, normativity, competence, sensitivity, continuity of attachment outcomes, reciprocity or bi-directionality of attachment, and psychopathology in childhood, adolescence, and adulthood. The attachment theory is universal. That means all assumptions of the attachment theory are available in all cultures and settings including normal and abnormal settings. This study focused on two assumptions measured in two distinctive groups, yet the remainder of the assumptions requires further investigation. The longitudinal study should be taken in consideration to examine the continuity assumption of the attachment outcomes in the non-Western cultural context.

## 5. Conclusions

In summary, using an innovative study design that includes psychiatric and non-psychiatric samples combined with a comprehensive analysis allows for the creation of a unique method by which the validity of universality and normativity assumptions related to the attachment theory in the context of a non-Western culture can be examined. This study confirms the validity of universality and normativity assumptions of the attachment theory in a non-Western culture.

## Figures and Tables

**Table 1:** Hazan and Shaver’s secure, avoidant, anxious/ambivalent categories.

**[Avoidant]** I am somewhat uncomfortable being close to others; I find it difficult to trust them completely, difficult to allow myself to depend on them. I am nervous when anyone gets too close, and often, love partners want me to be more intimate than I feel comfortable being.
**[Anxious-Ambivalent]** I find that others are reluctant to get as close as I would like. I often worry that my partner doesn’t love me or won’t want to stay with me. I want to get very close to my partner, and this sometimes scares people away.
**[Secure]** I find it relatively easy to get close to others and am comfortable depending on them. I don’t often worry about being abandoned or about someone getting too close to me.

**Table 2:** Skewness, Kurtosis, and Descriptive Statistics of Attachment Models.

Parameters	Adult Attachment Styles				Attachment Dimensions		Psychosis Attachment	
	Secure	Dismissing	Preoccupied	Fearful	Anxiety	Avoidance	Anxiety	Avoidance
	**Psychiatric Sample**							
**Skewness <1**	−0.221	−0.115	−0.196	−0.010	0.149	−0.047	−0.023	0.015
**Kurtosis <1**	−0.331	−0.414	−0.413	−0.797	0.026	0.189	−0.140	−0.349
**Mean**	15.84	14.58	12.34	11.77	71.76	57.14	18.33	18.79
**Median**	16.00	15.00	13.00	12.00	72.00	57.00	19.00	19.00
**Mode**	18.00	14.00	13.00	14.00	78.00	54.00	20.00	23.00
	**Non-Psychiatric Sample**							
**Skewness <1**	−0.024	−0.112	−0.039	0.355	0.066	−0.128	0.326	0.057
**Kurtosis <1**	−0.164	−0.369	−0.366	0.007	−0.397	0.131	−0.064	−0.378
**Mean**	15.40	14.70	11.75	10.96	70.92	57.07	16.93	18.96
**Median**	15.00	15.00	12.00	11.00	71.00	57.00	17.00	19.00
**Mode**	14.00	17.00	13.00	10.00	69.00	55.00	17.00	18.00

**Table 3:** Results of Various Rotation and Extraction Methods for Attachment Models Variables and their Factor Loadings Mean.

Attachment Variables	Seven Methods of Varimax							Seven Methods of Oblimin							Loadings Mean
	PCA	ULS	GLS	ML	PAF	AF	IF	PCA	ULS	GLS	ML	PAF	AF	IF	
**Psychiatric Sample**															
	**Adult Attachment Styles—RSQ “Method 1”**														
Secure	0.594	0.445	0.429	0.406	0.447	0.490	0.384	0.568	0.487	0.476	0.457	0.489	0.521	0.358	0.468
Dismissing	0.780	0.726	0.728	0.738	0.723	0.714	0.482	0.790	0.730	0.734	0.744	0.728	0.719	0.502	0.703
Preoccupied	0.691	0.556	0.571	0.545	0.556	0.557	0.436	0.687	0.578	0.592	0.569	0.578	0.579	0.439	0.567
Fearful	0.703	0.507	0.516	0.513	0.509	0.491	0.389	0.730	0.504	0.514	0.512	0.506	0.488	0.422	0.522
	**Attachment Dimensions—ECRS “Method 2”**														
Anxiety-ECRS	0.870	0.987	0.986	0.986	0.909	0.972	0.513	−0.882	−0.995	0.990	0.990	−0.921	0.982	−0.555	0.895
Avoidance-ECRS	0.854	0.534	0.549	0.551	0.572	0.536	0.504	−0.871	−0.570	0.583	0.582	−0.606	0.575	−0.552	0.602
	**Psychosis Attachment—PAM “Method 3”**														
Anxiety-PAM	0.889	0.999	0.995	0.996	0.849	0.848	0.523	0.903	−0.996	0.995	0.995	−0.852	0.848	−0.549	0.874
Avoidance-PAM	0.858	0.547	0.554	0.555	0.641	0.647	0.512	0.863	−0.567	0.570	0.570	−0.659	0.668	−0.524	0.624
**Non-Psychiatric Sample**															
	**Self-Model—Anxiety “Trait 1”**														
Anxiety-ECRS	0.780	0.746	0.992	0.994	0.751	0.784	0.592	−0.757	0.777	0.989	0.988	0.783	0.819	0.651	0.815
Anxiety-PAM	0.701	0.655	0.475	0.488	0.650	0.614	0.523	−0.671	0.686	0.577	0.570	0.682	0.612	0.584	0.606
Secure	0.731	0.467	0.396	0.398	0.468	0.472	0.389	−0.766	0.467	0.420	0.421	0.469	0.501	0.404	0.484
	**Other-Model—Avoidance “Trait 2”**														
Avoidance-ECRS	0.599	0.473	0.395	0.402	0.476	0.471	0.418	0.571	0.540	0.517	0.528	0.548	0.398	0.526	0.490
Avoidance-PAM	0.490	0.338	0.631	0.555	0.345	0.388	0.343	0.460	0.399	0.651	0.583	0.409	0.450	0.429	0.462
Dismissing	0.817	0.758	0.480	0.480	0.744	0.710	0.433	0.879	0.743	0.495	0.496	0.727	0.775	0.441	0.641
Preoccupied	0.565	0.443	0.423	0.429	0.447	0.419	0.373	0.545	0.495	0.492	0.500	0.501	0.350	0.453	0.459
Fearful	0.659	0.454	0.574	0.608	0.462	0.502	0.400	0.667	0.494	0.588	0.621	0.503	0.469	0.451	0.532

PCA: Principal Component Analysis, ULS: Unweight Least Square, GLS: Generalized Least Squares, ML: Maximum Likelihood, PAF: Principal Axis Factoring, AF: Alpha Factoring, IF: Image Factoring.

## Data Availability

The data used in the current study are confidential and cannot be publicly shared. This was also stated in the participants’ consent. However, it is available from the corresponding author on reasonable request.
